# Role of apurinic/apyrimidinic nucleases in the regulation of homologous recombination in myeloma: mechanisms and translational significance

**DOI:** 10.1038/s41408-018-0129-9

**Published:** 2018-09-25

**Authors:** Subodh Kumar, Srikanth Talluri, Jagannath Pal, Xiaoli Yuan, Renquan Lu, Puru Nanjappa, Mehmet K. Samur, Nikhil C. Munshi, Masood A. Shammas

**Affiliations:** 1 Dana Farber Cancer Institute, Boston, MA 02215 USA; 2 Veterans Administration Boston Healthcare System, West Roxbury, MA 02132 USA; 3 Multi-disciplinary Research Units, Pt J.N.M. Medical College, Raipur, CG India; 4 Harvard Medical School, Boston, MA 02215 USA

## Abstract

We have previously reported that homologous recombination (HR) is dysregulated in multiple myeloma (MM) and contributes to genomic instability and development of drug resistance. We now demonstrate that base excision repair (BER) associated apurinic/apyrimidinic (AP) nucleases (APEX1 and APEX2) contribute to regulation of HR in MM cells. Transgenic as well as chemical inhibition of APEX1 and/or APEX2 inhibits HR activity in MM cells, whereas the overexpression of either nuclease in normal human cells, increases HR activity. Regulation of HR by AP nucleases could be attributed, at least in part, to their ability to regulate recombinase (RAD51) expression. We also show that both nucleases interact with major HR regulators and that APEX1 is involved in P73-mediated regulation of RAD51 expression in MM cells. Consistent with the role in HR, we also show that AP-knockdown or treatment with inhibitor of AP nuclease activity increases sensitivity of MM cells to melphalan and PARP inhibitor. Importantly, although inhibition of AP nuclease activity increases cytotoxicity, it reduces genomic instability caused by melphalan. In summary, we show that APEX1 and APEX2, major BER proteins, also contribute to regulation of HR in MM. These data provide basis for potential use of AP nuclease inhibitors in combination with chemotherapeutics such as melphalan for synergistic cytotoxicity in MM.

## Introduction

Most cancers display a variety of genomic alterations at diagnosis^1–6^ and acquire new changes with progression of disease to advanced stages^7–10^ indicating an instability of genome^1,11–17^. We have observed a similar pattern of change in multiple myeloma (MM), both at diagnosis and in relapse samples^1^. The ability to constantly evolve not only enables the MM cells to acquire new characteristics for growth, survival, and progression of disease, but also presents a great obstacle to treatment. Understanding mechanisms underlying genomic instability is therefore essential to develop effective strategies for prevention and treatment of cancer.

Normal cellular DNA is subject to a large number of chemical and physical modifications^18,19^ which may include missing or altered bases, deletions/insertions, DNA strand breaks and cross-linking etc. Therefore, multiple DNA repair mechanisms exist and ensure that these alterations are constantly and efficiently repaired in normal cells. Homologous recombination (HR), which depends on sequence homology/identity, is one of the most precise DNA repair mechanism^20^. HR is involved in the repair of a variety of DNA damage types including DNA double-stranded breaks, DNA strand crosslinks and single-stranded DNA gaps, and plays a vital role in the maintenance of genomic integrity^21^. However, dysregulation of HR can also lead to genomic instability^22^. Consistent with this, we have previously demonstrated that elevated HR mediates genomic instability and progression in MM^23^. Using another cancer model system (Barrett’s adenocarcinoma), we demonstrated that dysregulated HR disrupts genomic integrity^24^ and stability^25^, and transgenic as well as chemical suppression of HR, significantly reduced both the genomic instability as well as growth of cancer cells in mice^26^. Identification of mechanisms underlying dysregulation of HR thus may help prevent genomic evolution and provide novel targets for cancer prevention.

Investigating mechanisms underlying dysregulation of HR, we identified significant role for apurinic/apyrimidinic (AP) nucleases (APEX1 and APEX2). APEX1 and APEX2 are the key base excision repair (BER) pathway proteins involved in the recognition and repair of AP (or abasic) sites^27–30^ and play a significant role in genome maintenance. Development of abasic site, one of the most common DNA damage, can occur either spontaneously or by removal of a damaged DNA base by specific enzymes, DNA glycosylases. The repair of AP sites is extremely efficient. APEX1 and APEX2 are major BER proteins involved in its initiation. APEX1 is the abasic endonuclease implicated in both the BER and 3′-end processing in mammalian cells^29,31,32^. It is a multifunctional protein and is also responsible for repair of alkylation and oxidative DNA damage in a cell. The protein also functions as a reduction–oxidation (redox) factor and hence maintains many transcription factors in an active reduced state^33,34^. APEX1 is a strong endonuclease but also has a weak 3′-5′ exonuclease activity for proofreading function^35,36^. APEX2 on the other end has a weak endonuclease but a strong exonuclease activity^35^. Elevated levels of APEX1 have been found in cervical^37^, ovarian^38,39^, prostate^40^, germ cell tumors^41^, and correlate with the radiosensitivity of cervical cancers^42^. Here we have explored the role of APEX1 and APEX2 in dysregulation of HR in MM.

## Materials and methods

### Cell lines and patient samples

MM cell lines RPMI8226 and U266 were purchased from the American Type Tissue Culture Collection (Rockville MD), ARP was a gift from Dr. J. Epstein (University of Arkansas for Medical Sciences) and MM.1S obtained from Dr. Steven Rosen (Northwestern University, Chicago, IL). Cell lines, confirmed to be negative for mycoplasma, were cultured in RPMI1640 medium containing 10% fetal bovine serum (HyClone, South Logan, UT) as described previously^43–49^ and maintained in logarithmic growth.

Bone marrow aspirate samples from normal donors and patients were obtained following informed consent under protocol approved by Institutional Review Board, Dana Farber Cancer Institute. Plasma cells were isolated by Magnet Assisted Cell Sorting (MACS, Miltenyi Biotech) according to the manufacturer’s protocol, and their purity (>95%) confirmed by monitoring cell surface expression of CD45 and CD38^50^.

### Reagents

Anti-APEX1 antibody, catalogue # NB100-101 was purchased from Novus Biologicals, Inc., (Littleton, CO). Anti-APEX2 antibody is developed by us. Anti-RAD51 antibody, catalogue # SC-8349 was purchased from Santa Cruz, Dallas, TX. Antibodies against GAPDH (catalogue # mAb2118), HP1 (catalogue # 261), and γH2A.X (Ser139, catalogue #2577) were from Cell Signaling Technology, Inc., Danvers, MA. For immunoprecipitation studies, anti-APEX1 antibody (Cat. # ab194, Abcam) and anti-p73 antibody (Cat. # NBP2-24737, Novus Biologicals) were used.

### Modulation of AP nuclease expression

AP nuclease expression was modulated using chemical as well as transgenic manipulations. AP nuclease activity was suppressed using APEX1 inhibitor (APE1 Inhibitor III; API3) (Axon Medchem LLC, Reston, VA), methoxyamine (MX) which inhibits AP nuclease activity by binding to aldehyde group of abasic site (Sigma Aldrich, Saint Louis, MO) and lentivirus based shRNAs targeting APEX1 and APEX2 (Sigma Aldrich, Saint Louis, MO). For overexpression, the plasmids carrying these genes under CMV promoter were used.

### AP nuclease activity assay

AP nuclease activity was assessed using a fluorescence based assay reported previously^51^. Briefly, the oligonucleotides carrying AP site mimic (Tetrahydrofuran; THF) and a fluorescein label on one strand and a quenching moiety (Dabcyl-Q) on other strand are synthesized commercially (Eurogentec Ltd., San Diego, CA). The AP nuclease activity cleaves AP site releasing the short fluorescein-labeled fragment, thus resulting in the increase in fluorescence.

### Recombination assays

Three different functional assays were used to evaluate HR in this study. A plasmid based HR activity assay has been reported previously^25,26^. Here, we also used a modification of this assay in which HR substrate plasmid is mixed with MM cell lysate under in vitro HR conditions^52^, and product of HR measured by Q-PCR. This modified version of HR assay was validated and was consistent with the assay conducted within intact cells (Supplementary Figure 1). We also used a fluorescence based homologous strand exchange (SE) assay to measure HR, as reported by^53^.

### Detection of DNA breaks

DNA breaks were estimated by evaluating MM cells for levels of γ-H2AX. Expression of γ-H2AX was measured by Western blotting using anti-H2AX (Ser139, antibody # 2577; Cell Signaling Technology, Inc., Danvers, MA) as reported by us previously^24^.

### Western blotting and immunocytochemical detection of proteins

For western blotting, the proteins fractionated on gradient SDS-acrylamide gel were electroblotted onto nitrocellulose paper and sequentially treated with indicated primary antibodies and either anti-rabbit or anti-mouse horseradish peroxidase (HRPO) conjugates. Blots were then washed and specific proteins detected using an enhanced chemiluminescence, according to the instructions provided by manufacturer (Amersham Life Sciences Inc., Arlington Heights, IL). For immunocytochemical detection of APEX1, cytospins of normal plasma cells and MM cells were fixed in methanol/acetone (1:1, v/v) for 10 min at –20 °C. Fixed cells were rinsed, rehydrated in PBS, and incubated with antibody to APEX1. Vectastain Elite ABC kit (Vector Laboratories, Burlingame, CA) was used for immunostaining as described by the manufacturer.

### Chromatin immunoprecipitation and co-immunoprecipitation

Chromatin immunoprecipitations were performed using EZ-Magna ChIP™ A/G Chromatin Immunoprecipitation Kit (Millipore). Cells were fixed in formaldehyde (1%) for 10 min at room temperature. Glycine was then added to a final concentration of 0.125 M to quench the reaction. Cells were washed twice in cold PBS and once with PBS containing protease inhibitors, incubated in lysis buffer with protease inhibitors on ice for 15 min, and centrifuged at 800 × *g* for 5 min at 4 °C. The supernatant was removed and the nuclear pellets re-suspended in nuclear lysis buffer with protease inhibitors and incubated on ice for 10 min before sonication. Sonication was performed using a probe sonicator in 1.5 ml tubes. Sonication conditions involved 11 cycles at 75% amplitude, 30 s pulse, 1 min on ice, followed by 1 cycle at 80% amplitude. The lysates were centrifuged at 12,000 × *g* for 10 min at 4 °C and the chromatin supernatants were used for immunoprecipitation, using 4 µg of APEX1 antibody (catalogue # ab194, Abcam) or p73 antibody (catalogue # NBP2-24737, Novus Biologicals) or IgG controls. Real-time PCR reactions, using primers for amplification of a region of RAD51 promoter, were conducted to assess the fraction of RAD51 promoter DNA associated with proteins under investigation. Primers specific to GAPDH promoter region were used as negative control.

### Investigation of protein–protein interactions, expression, and phosphorylation levels

For investigation of direct protein–protein interactions, a cell cycle antibody array (Hypromatrix, Worcester, MA) containing 60 antibodies, and/or a custom antibody array having 40 antibodies immobilized on a nitrocellulose membrane, were sequentially incubated with MM cell lysates and then HRP-conjugated anti-APEX1 or APEX2 antibodies. Interacting partners were then identified by their address on the array.

### Evaluating impact on micronuclei, a marker of genomic instability

To evaluate impact on genomic instability, the cells were cultured in the presence or absence of inhibitor of AP nuclease activity (API3), melphalan or combination of both, and cells evaluated for micronuclei, a marker of unstable genome^54,55^, using Micronucleus Assay MicroFlow kit (Litron Laboratories, New York, USA).

### Statistical analyses

**Fig. 1:**
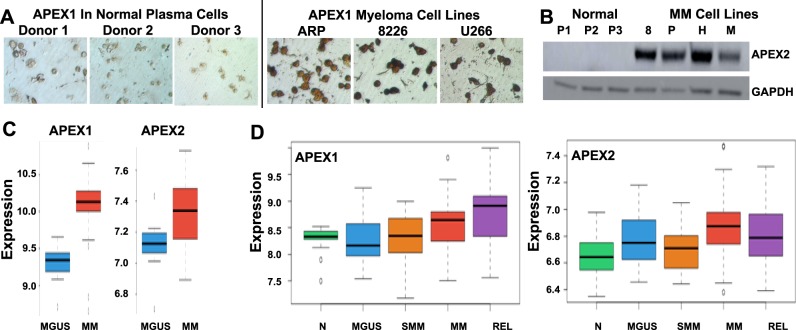
**AP nuclease expression is elevated in MM.** **a**-**b.** Protein levels of APEX1 and APEX2 are elevated in MM cells; **a.** Immunocytochemistry showing APEX1 in normal samples (Donors 1–3) and MM cell lines; **b.** Western blot showing APEX2 expression in normal and MM cell lines; **c.** Relative expression (Log2) of APEX1 and APEX2 in 7 MGUS and 39 MM samples from GSE2113 dataset were computed using modified *t* test in limma. **d.** Relative expression (Log2) of APEX1 and APEX2 in GSE6477 dataset (*N* = 16, MGUS = 22, SMM = 24, MM = 73 and Relapse = 28) plotted using R and normal and MM samples compared with limma

For evaluation of APEX1 and APEX2 expression in MM datasets, expression dataset (GSE2113; Fig. 1c) and (GSE6477; Fig. 1d) were downloaded from GEO (https://www.ncbi.nlm.nih.gov/geo/) database and expression profiles pre-processed with R using affy and limma packages for Affymetrix Human Genome U133A Array. RMA normalization was applied to both datasets. 7 MGUS and 39 MM samples from GSE2113 were compared using modified *t* test in limma. Expression levels in GSE6477 dataset (*N* = 16, MGUS = 22, SMM = 24, MM = 73, and Relapse = 28) was plotted using R and normal and MM samples were compared with limma. For gene array (Supplementary Figure 2), RNA derived from untreated and methoxyamine treated ARP cells was evaluated using HG-U133 array (Affymetrix); Arrays were normalized and expression values calculated, using DNA-Chip Analyzer, as described previously ^23,44,45^.

## Results

### APEX1 and APEX2 are overexpressed in MM

In our previous study, evaluation of gene expression by microarray indicated that expression of APEX1 and/or APEX2 in MM cell lines and a subset of MM patient samples was upregulated relative to normal plasma cell samples^23^. To further investigate AP nucleases in MM, here we evaluated their expression at protein level using immunocytochemistry (for APEX1) and western blotting (for APEX2) and confirmed upregulation in MM relative to normal plasma cells (Fig. 1a, b). To further investigate AP nuclease expression in other MM datasets, expression datasets (GSE2113, GSE6477) were downloaded from GEO (https://www.ncbi.nlm.nih.gov/geo/) database and expression profiles pre-processed with R using affy and limma packages for Affymetrix Human Genome U133A Array. RMA normalization was applied to both datasets. 7 MGUS and 39 MM samples from GSE2113 were compared using modified *t* test in limma. APEX1 was 1.8 fold upregulated in MM compared to MGUS (*p* value < 1e-4) and APEX2 was 1.2 fold upregulated in MM (*p* value = 0.047) (Fig. 1c). Expression levels in GSE6477 dataset (*N* = 16, MGUS = 22, SMM = 24, MM = 73, and Relapse = 28) were plotted using R and normal and MM samples compared with limma. Both APEX1 and APEX2 were upregulated with *p* values 0.02 and 1e-3, respectively (Fig. 1d).

### Elevated AP nuclease expression dysregulates HR in MM cells

**Fig. 2:**
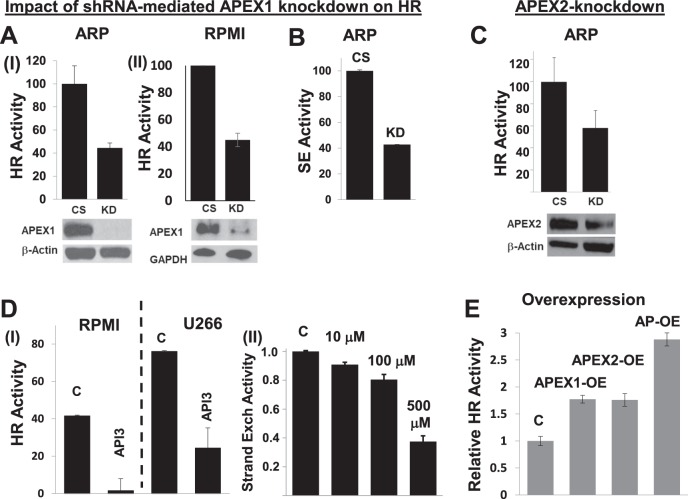
**AP nucleases regulate HR activity in MM cells.** MM cells were subjected to various transgenic or chemical manipulations to inhibit APEX1 and/or APEX2, and cells evaluated for HR activity. **a.** MM cell lines (ARP and RPMI8226), transduced with lentivirus particles carrying control shRNA (CS) or APEX1-shRNA (KD), were selected in puromycin and evaluated for APEX1 expression by Western blotting (bottom panels) and HR activity using a plasmid based in vitro HR assay (top panels); error bars represent SDs of triplicate assays. **b.** Control and APEX1-knockdown ARP cells shown in panel A were also evaluated for HR using a different assay which measures homologous strand exchange (SE), a critical step in HR. **c.** MM cells (ARP), transduced with lentivirus particles carrying control shRNA (CS) or APEX2-shRNA (KD), were selected in puromycin and evaluated for APEX2 expression by western blotting (bottom panel) and HR activity using a plasmid based in vitro HR assay (top panel); error bars represent SDs of triplicate assays. **d.** (panel I) MM cell lines (RPMI and U266) were treated with DMSO (C) or APEX1 inhibitor (API3; 5 μM) for 24 h and evaluated for impact on HR; (panel II) ARP cells were treated with various concentrations of AP nuclease inhibitor methoxyamine (MX) for 48 h and impact on HR evaluated. **e.** Normal fibroblast cells transfected with control plasmid (C) or those for overexpression of APEX1 (APEX1-OE), APEX2 (APEX2-OE) or both AP nucleases (AP-OE) were evaluated for HR activity in intact cells using plasmid based assay^25,26^; error bars represent SEMs of triplicate assays

To investigate the functional role of elevated APEX 1/2 on various cellular processes, we evaluated the impact of chemical inhibition of AP nuclease activity with mithoxyamine (MX) and identified prominent perturbation of HR-related genes (Supplementary Figure 2; full microarray data submitted as Supplementary Table 1). We also observed that the expression of APEX1 and APEX2 in MM patient dataset (gse26863) correlated with the expression of a panel of HR genes (Supplementary Figure 3) identified in a functional screen^56^. Therefore, we next investigated the impact of transgenic and chemical modulations of APEX1 and/or APEX2 on HR activity, using three different functional HR assays; measuring HR activity by transfecting a plasmid substrate into cell^25,26^; utilizing the same plasmid under in vitro HR assay conditions^52^; and by measuring homologous strand exchange (SE) activity^53^, an essential step in the initiation of HR. APEX1-knockdown inhibited HR activity in ARP and RPMI8226 MM cells as measured by in vitro HR assay (Fig. 2a) as well as by SE assay in ARP (Fig. 2b) and RPMI8226 (data not shown) cells. Similarly, shRNA-mediated knockdown of APEX2 also inhibited HR activity in ARP cells (Fig. 2c). Treatment of MM cell lines (RPMI8226 and U266) with a small molecule inhibitor of APEX1 (API3) also inhibited HR activity in these cells (Fig. 2d, panel I). Additionally, the treatment of ARP MM cells with methoxyamine (MX) an inhibitor of overall AP activity by binding to aldehyde group of abasic sites led to a dose-dependent inhibition of HR activity, as measured by SE assay (Fig. 2d**, panel II**). Consistent with these data, the upregulation of AP nuclease in normal human (fibroblast) cells, separately and in combination with each other, increased HR activity by ~1.8-fold and ~3-fold (*p* > 0.05), respectively (Fig. 2e). Taken together, these data confirm that APEX1 and APEX 2 are involved in regulation of HR, and their elevated expression may at least in part contribute to dysregulation of HR activity in MM cells.

### **AP nucleases regulate RAD51 expression in MM cells.**

**Fig. 3:**
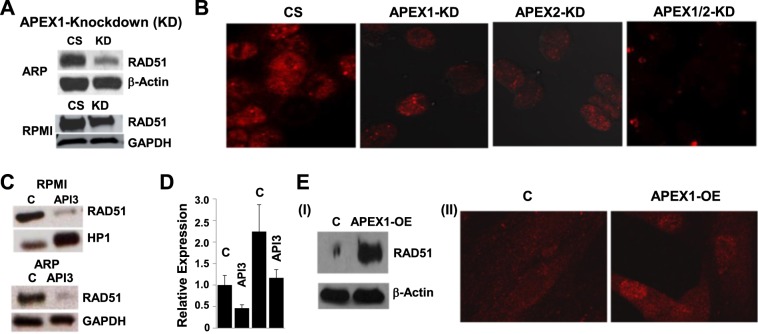
**AP nucleases regulate RAD51 expression in MM cells.** **a.** Suppression of APEX1 inhibits RAD51 protein. Control and APEX1-knockdown ARP and RPMI8226 cells (for which APEX1 knockdown is shown in Fig. 2a, bottom) were evaluated for impact on RAD51 expression using western blotting. CS, control shRNA; KD, APEX1-knockdown; **b.** RAD51 foci in control (CS) ARP cells or those with knockdown (KD) of APEX1, APEX2 or both these genes (APEX1/2-KD); **c.** RPMI8226 and ARP cells, control and treated with AP inhibitor A PI3 (5 μM for 24 h) were evaluated for RAD51 by western blotting; **d.** Inhibition of APEX1 inhibits transcript levels of RAD51. RPMI8226 cells were treated with API3 (5 μM) for 24 h and transcript levels of RAD51 evaluated by quantitative RT-PCR; error bars indicate SDs of triplicate assays. **e.** Western blot showing RAD51 (I) and RAD51 foci in normal fibroblast cells, control (C) and APEX1-overexpressing (APEX1-OE)

To further investigate how AP nucleases impact HR activity, we evaluated AP-knockdown cells for their effect on RAD51 recombinase expression as well as RAD51 foci formation. As seen in Fig. 3a, APEX1-knockdown (KD) inhibits RAD51 expression in ARP and RPMI8226 MM cell lines (panel a). Consistent with these observations, the knockdown of APEX1, APEX2 or both inhibited RAD51 focus formation in ARP cells (Fig. 3b). Similarly, the small molecule inhibitor of AP activity (API3) also inhibited RAD51 expression in RPMI8226 and ARP (Fig. 3c) cells associated with reduction in RAD51 transcript (Fig. 3d), suggesting a transcription level regulation. As expected, the transgenic upregulation of APEX1 in normal human fibroblast cells increased RAD51 expression as well as RAD51 foci (Fig. 3e). These data demonstrate that APEX1 and APEX2 in MM cells are involved in the regulation of RAD51, the protein which mediates homologous pairing, a key step in HR.

### APEX1 is involved in p73-mediated transcriptional regulation of RAD51 in MM cells

**Fig. 4:**
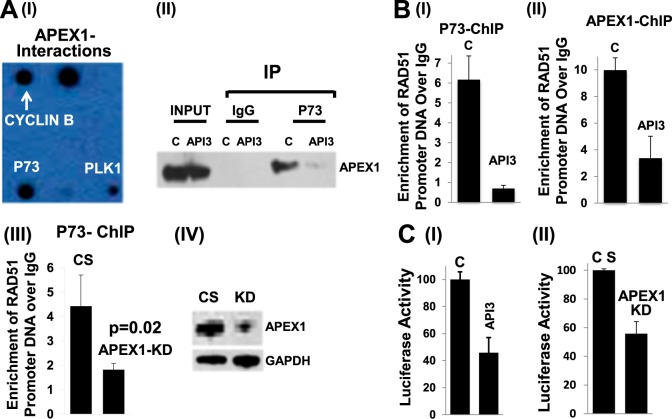
**APEX1 is involved in P73-mediated regulation of RAD51.** **a** (I) Antibody array**:** Cell Cycle Antibody Array (Hypomatrix) was sequentially treated with ARP cell lysate and HRP-conjugated anti-APEX1 antibody. Interacting partners were then identified by their location on the array. (II) Immunoprecipitation: Interaction between endogenous APEX1 and p73 was detected in the ARP cells, either control (DMSO) or those treated with 5 µM API3 for 24 h. Nuclear lysates were immunoprecipitated using anti-P73 antibody or IgG (control) and protein complexes resolved on Western blot which was probed with anti-APEX1 antibody; Input, lysate before immunoprecipitation. **b** (I-III): APEX1 and P73 bind to RAD51 promoter in MM cells. Protein-DNA complexes from ARP cells treated as below were immunoprecipitated using anti-P73 antibody (I and III) or anti-APEX1 antibody (II). DNA was then extracted and occupancy of RAD51 promoter evaluated by Q-PCR; Treatments**:** C, control (DMSO); API3, cells treated with APEX1 inhibitor 5 µM for 24 h; CS, control shRNA; APEX1-KD, APEX1-knockdown cells; (IV) Western blot showing APEX1 in CS and APEX1-KD cells. **c**. Inhibition of APEX1 inhibits RAD51 promoter activity in MM cells. (I) RAD51 promoter was cloned upstream of firefly luciferase gene in a plasmid, which was then introduced into ARP cells. APEX1 in these cells was inhibited by treating them with small molecule (API3, 5 µM) for 24 h, and cells evaluated for luciferase activity; (II) ARP cells transduced with lentivirus particles carrying control (CS) or APEX1-shRNA (APEX1-KD) were selected in puromycin and following confirmation of knockdown, cells were co-transfected with RAD51 promoter plasmid (described in panel I) and a plasmid carrying Gaussia luciferase as control for transfection efficiency. RAD51 promoter activity was assessed from ratio of two luciferase activities; error bars represent SDs of triplicate assays

To further investigate how AP nuclease regulates RAD51, we investigated the interacting partners of APEX1 and APEX2 using standard as well as custom-synthesized antibody arrays (Supplementary Figure 4). We observed that both APEX1 (Fig. 4a-I; Supplementary Figure 4A) and APEX2 (Supplementary Figure 4B) interact with a number of HR-related proteins. Interestingly, both antibody arrays (Fig. 4a-I; Supplementary Figure 4A) and co-immnoprecipitation experiment (co-IP; Fig. 4a-II) identified APEX1 interaction with P73, a known transcriptional regulator of RAD51. Furthermore, a chromatin immunoprecipitation (ChIP) assay showed that both APEX1 and P73 bind to RAD51 promoter in MM cells; and inhibition of APEX1 by small molecule inhibitor or shRNA-mediated APEX1 knockdown, inhibits the association of P73 with RAD51 promoter (Fig. 4b I-IV). To further investigate the role of APEX1 in transcriptional regulation of RAD51, we transfected MM cells with a plasmid carrying luciferase gene under RAD51 promoter. APEX1 in these cells was then inhibited either by API3 or APEX1-shRNA, and luciferase activity assessed. APEX1 inhibition, by either method was able to inhibit RAD51 promoter activity in MM cells (Fig. 4c I-II). Taken together, these data suggest that APEX1 is involved in P73-mediated regulation of RAD51 expression in MM cells.

### Elevated AP expression contributes to melphalan-resistance in MM

**Fig. 5:**
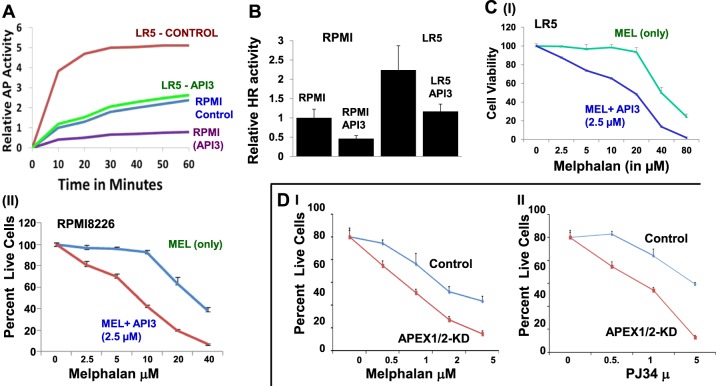
**AP activity contributes to melphalan resistance and suppression of AP nucleases increases melphalan-induced cytotoxicity in MM cells.** **a.** AP nuclease activity was measured in parental RPMI8226 and melaphalan-resistant LR5 cell lines in the presence or absence of AP nuclease inhibitor API3 (2.5 µM) as described in 'Methods'; time-dependent change in AP activity is shown. **b.** HR activity measured in parental RPMI8226 and melaphalan-resistant LR5 cell lines in the presence or absence of AP nuclease inhibitor API3 (2.5 µM) as described in 'Methods'; error bars represent SDs of triplicate assays. **c.** LR5 (I) and RPMI8226 (II) cells were treated with different concentrations of melphalan (MEL) alone or in combination with 2.5 µM of APEX1 inhibitor (API3) for 48 h and cell viability measured. **d.** AP (APEX1/2-KD) knockdown increases sensitivity of MM cells to melphalan and PARP inhibitor: **d.** Control (CS) ARP cells or those in which both APEX1 and APEX2 were suppressed (APEX1/2-KD; same cells as shown in Fig. 3b) were treated with different concentrations of melphalan (I) or PARP inhibitor, PJ34 (II) and cell viability measured by Cell Titer Glow for 7 days

We have previously shown that increased HR activity contributes to development of drug resistance in MM^23^. Since AP nuclease expression regulates HR activity, we investigated its role in melphalan resistance in MM cells. RPMI8226 and melphalan-resistant MM cells (LR5) were treated with API3 (2.5 μM) and impact on AP and HR activities monitored as described. Both AP and HR activities, although higher in resistant LR5 relative to melphalan-sensitive RPMI8226 cells, were inhibited ∼50% by API3 in both cell lines (Fig. 5a, b). Moreover, AP nuclease inhibitor sensitized both sensitive and resistant MM cell lines to melphalan treatment (Fig. 5c). Importantly, the dose of melphalan (20 μM) which had almost no effect on viability of resistant LR5 cells, induced >50% cell death in the presence of AP nuclease inhibitor (Fig. 5c, panel I). To further investigate if inhibition of AP nuclease expression increases efficacy of melphalan in MM, we suppressed both APEX1 and APEX2 in ARP cells and observed sensitization of MM cells to both melphalan and a PARP inhibitor PJ34 (Fig. 5d).

### Inhibition of AP activity increases melphalan-induced cytotoxicity while reducing DNA damage and instability

**Fig. 6:**
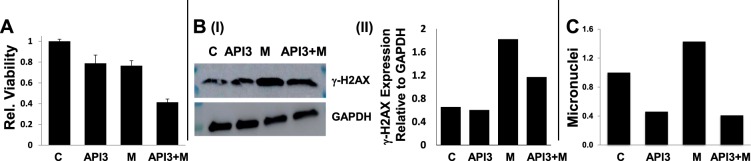
AP nuclease inhibitor increases melphalan-induced cytotoxicity but reverses genomic instability caused by it. MM (RPMI) cells, control (C) or those treated with melphalan (M), AP nuclease inhibitor (API3; 1 μM) or combination (API3-M) were evaluated for cell viability (**a**), DNA breaks by evaluating γH2AX expression (a DNA break marker) by western blotting (**b**) and genomic instability as assessed from micronucleus assay (**c**). Error bars in panel A represent SDs of triplicate assays

To investigate impact of AP nuclease inhibitor on melphalan-induced cytototoxicity, we evaluated their effect on DNA damage and genomic instability. RPMI8226 cells were treated with API3 (at a lower dose of 1 μM), melphalan or combination of both for 48 h and evaluated for cell viability and genomic instability. As shown in Fig. 6, treatment with melphalan increased cell death and genomic instability in MM cells, whereas AP nuclease inhibitor, although increased melphalan-induced cell death (panel A), was able to reverse melphalan-induced DNA-damage (γ-H2AX) (panel B) and genomic instability (assessed by micronucleus assay) (panels C) in MM cells.

## Discussion

HR is a DNA repair mechanism that utilizes sequence homology in the participating DNA strands to copy missing information either from a sister chromatid (in the G_2_ phase) or from homologous chromosome (in G1 phase of cell cycle)^57^. Since availability and utilization of sister chromatid as template in G2 phase of cell cycle makes the process of HR much more accurate (or error free), the regulatory mechanisms ensure that HR in G1 is suppressed and mitotic recombination machinery uses sister chromatid instead of a homolog chromosome as a template to repair the damage^58^. Thus HR, especially associated with G2 phase of cell cycle, has a unique role in the maintenance of genomic integrity and stability by accurately repairing double-strand breaks in DNA. Moreover, if DNA breaks escape the repair system and persist from G_2_ into mitosis, they can recombine in G_1_ to produce gene rearrangements. In normal cellular environment, the process of HR is tightly regulated and precise. However, the process of HR which involves incision and recombination of genomic DNA fragments, can also be deleterious, if dysregulated or altered. Consistent with this, we have previously shown that elevated HR activity mediates genomic instability and development of drug resistance in MM^23^. Investigating the mechanisms underlying dysregulation of HR, here we have identified AP nucleases (APEX1 and APEX2) as novel regulators of HR in MM. Using multiple cell types and various approaches and evaluating various features of HR, we show that transgenic as well as chemical suppression of AP nuclease activity inhibits HR in MM cells, whereas transgenic upregulation of AP nucleases in normal cells increases HR activity. These data suggest that besides important roles in BER, AP nucleases also regulate HR in MM. These data are consistent with the observations in glioblastoma indicating the role of APEX1 in the regulation of HR^59^. However, our study further demonstrates that ability of both APEX1 and APEX2 to regulate HR can be partly attributed to their ability to regulate RAD51 expression in MM cells. We show that transgenic as well as chemical suppression of AP nucleases inhibits RAD51 expression in MM as well as esophageal cancer (not shown) cells, whereas transgenic upregulation of APEX1 as well as APEX2 induces RAD51 expression. An important finding here is that APEX1 is involved in the regulation of HR, through P73-mediated transcriptional regulation of RAD51. However, we do not rule out the possibility that APEX1 may also be involved in regulation of RAD51/HR by other mechanisms. Consistent with this view, we observe that in addition to P73, APEX1 also interacts with other HR regulators including BRCA1, P53, and probably RAD51 itself (Supplementary Figure 4A). Although association of APEX1 with P73, a transcriptional regulator of RAD51^60^, was confirmed, APEX2 did not show interaction with P73 but with several other HR regulators including BRCA1, BRCA2, BARD1, RAD52, and P53. These data suggest that although APEX1 and APEX2 have some unique interacting partners, in general, both nucleases interact with major HR regulators and interconnect HR with BER.

Consistent with the role of AP nuclease expression in HR activity, both AP and HR activities were higher in melphalan-resistant, relative to sensitive cells. Moreover, the inhibition of AP nuclease activity increased the melphalan cytotoxicity even overcoming melphalan-resistance in MM cells. These data further support the evidence that AP nuclease inhibitors can be combined with melphalan and possibly other chemotherapeutic agents to increase their cytotoxicity. In MM cells, melphalan increased cell death as well as genomic instability. However, addition of AP inhibitor, although increased melphalan-induced cytotoxicity, was able to reverse the genomic instability caused by melphalan. Since melphalan causes DNA breaks, the cells with extensive breaks undergo apoptosis. However, melphalan-treated cells which have limited breaks and survive apoptosis, may undergo HR-mediated repair which may expose them to genomic rearrangements and instability. This is evident by increase in micronuclei (Fig. 6) and also by observation that AP nuclease inhibitor increases melphalan-induced cytotoxicity but reverses associated genomic instability. Commensurate with these data, we have also observed that just like AP nuclease inhibitor, inhibitors of RAD51 also increase melphalan-induced cytotoxicity but reverse genomic instability associated with it (unpublished data from our laboratory). Our results therefore show that AP nucleases play an important role in HR through regulation of RAD51 expression, and their inhibitors have potential to inhibit growth as well as ability to inhibit/delay evolution of MM cells. These inhibitors also have ability to increase cytotoxicity of chemotherapeutic agents and potential to reduce their harmful genomic impact.

## Electronic supplementary material

Supplementary Figure Legends

Supplementary Figure 1

Supplementary Figure 2

Supplementary Figure 3

Supplementary Figure 4

Supplementary Figure 5

Supplementary Table 1
