# Supplementary material for: Role of apurinic/apyrimidinic nucleases in the regulation of homologous recombination in myeloma: mechanisms and translational significance
Source: Blood Cancer J. 2018 Sep 25;8(10):92. doi: 10.1038/s41408-018-0129-9 (PMC6177467; doi:10.1038/s41408-018-0129-9)
Supplement: Supplementary file 1 — Supplementary Figure Legends [file 41408_2018_129_MOESM1_ESM.docx]

**Supplementary Figures:**

**Supplementary Figure 1. Comparison of evaluation of HR activity in intact cells vs. in the cell lysates *in vitro*.** FLO-1 cancer cells were subjected to different transgenic and chemical manipulations, cells divided in two groups, one group processed for evaluation of HR activity following transfection of substrate plasmid reported previously (25, 26), whereas other group processed for evaluation of HR activity in the cell lysates.

**Supplementary Figure 2. Gene expression profiling of ARP cells treated with AP inhibitor (methoxyamine; MX):** ARP cells, untreated or treated with MX (500 µM) for 24 hrs, were evaluated for gene expression profiling using using HG-U133 array (Affymetrix). Colour scale at bottom of the figure shows percent change of expression in MX-treated, relative to control ARP cells. Complete microarray data is presented as Supplementary Table 1.

**Supplementary Figure 3.** Patients in MM dataset (gse26863) were divided into three groups based on AP nuclease expression levels, and expression of APEX1 (I) and APEX2 (II) evaluated for correlation with HR genes identified in a functional screen (57).

**Supplementary Figure 4. APEX1 and APEX2 both interact with major HR regulators.** Custom antibody arrays containing 40 different antibodies related to cell cycle and DNA repair proteins (Hypomatrix) were sequentially treated with RPMI cell lysate and HRP-conjugated anti-APEX1 or HRP-conjugated anti-APEX2 antibodies. Interacting partners of APEX1 (A) and APEX2 (B) were then identified by their location on the array.

**Supplementary Figure 5. AP nuclease inhibitor reverses melphalan-induced genomic instability in MM cells.** MM (RPMI8226) cells, control (C) or those treated with melphalan (M), AP inhibitor (API3; 1 μM) or combination (API3-M) were evaluated for genomic instability as assessed from micronucleus assay using flow cytometry. Images show changes in micronuclei fraction following these treatments. Bar graph of same is shown in Figure 6C.

**Supplementary Table 1. Microarray data showing impact of methoxyamine on genomewide expression profile of ARP cells.** RNA from ARP cells, untreated or treated with methoxyamine (MX; 500 µM) for 24 hrs, was evaluated for gene expression profiling using HG-U133 array (Affymetrix) and data analyzed as described in Methods.
