# Supplementary figures and images for: Role of apurinic/apyrimidinic nucleases in the regulation of homologous recombination in myeloma: mechanisms and translational significance
Source: Blood Cancer J. 2018 Sep 25;8(10):92. doi: 10.1038/s41408-018-0129-9 (PMC6177467; doi:10.1038/s41408-018-0129-9)

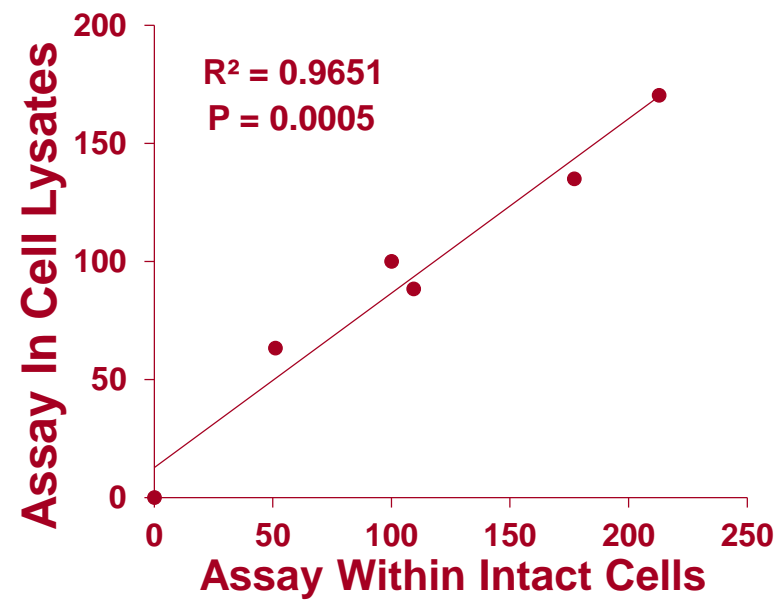

Supplement: Supplementary file 2 — Supplementary Figure 1 [file 41408_2018_129_MOESM2_ESM.pdf]

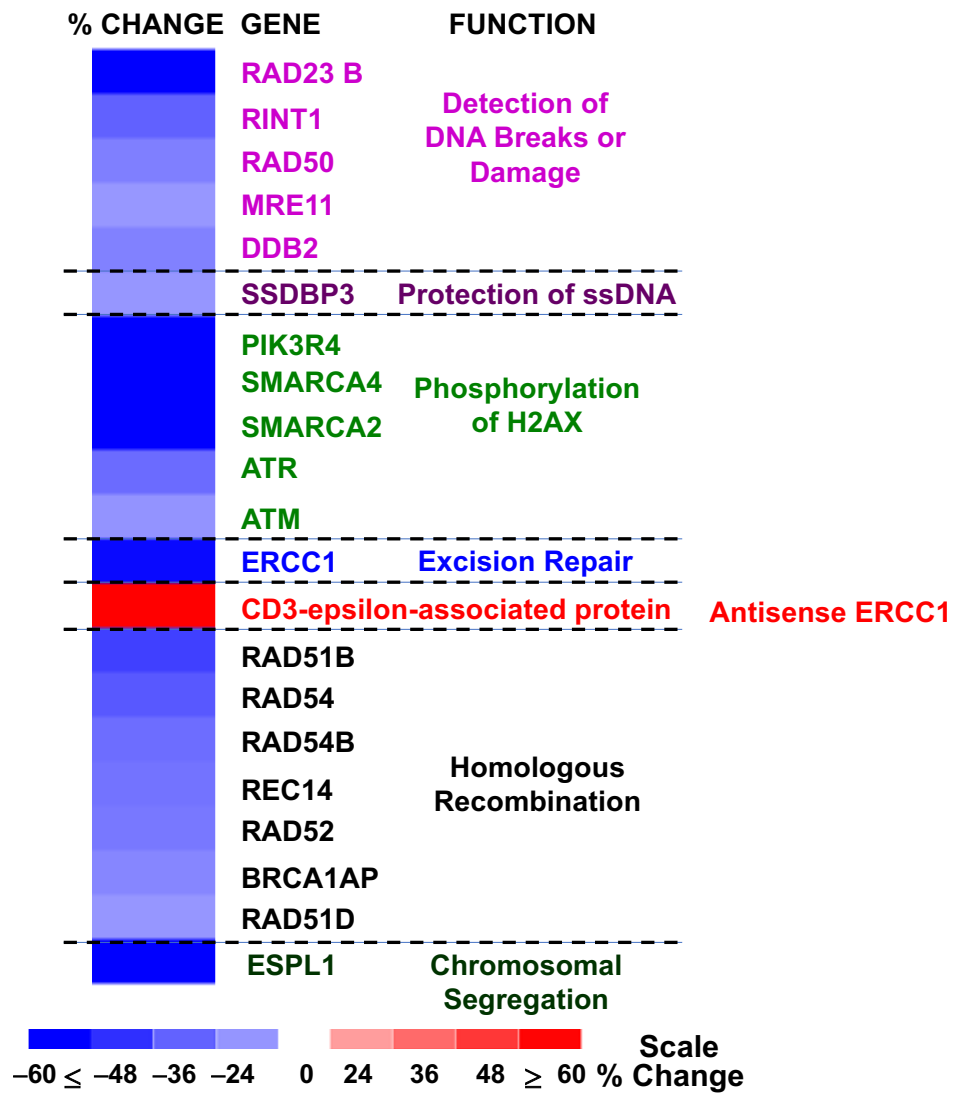

Supplement: Supplementary file 3 — Supplementary Figure 2 [file 41408_2018_129_MOESM3_ESM.pdf]

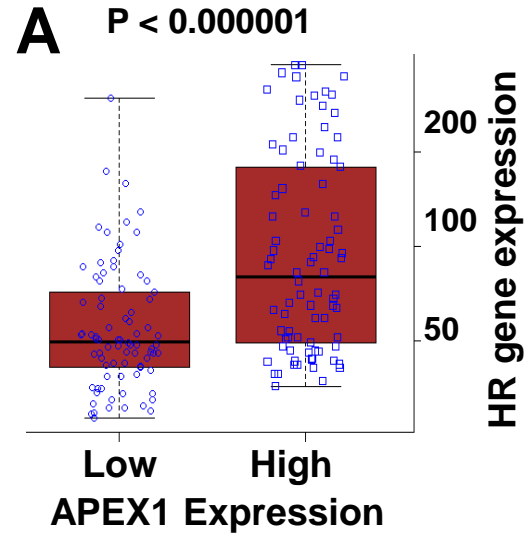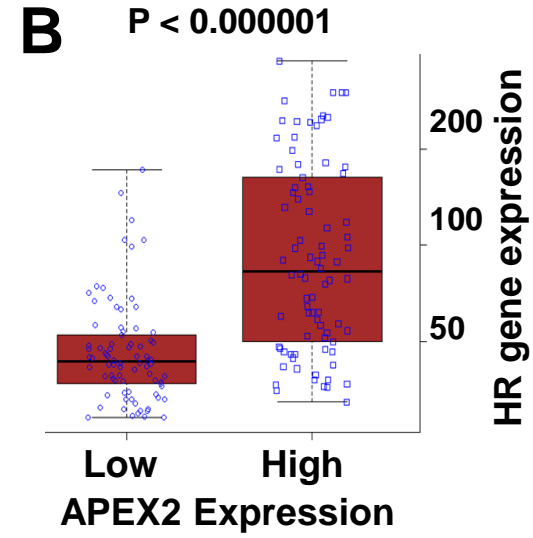

Supplement: Supplementary file 4 — Supplementary Figure 3 [file 41408_2018_129_MOESM4_ESM.pdf]

## A APEX1-interacting partners

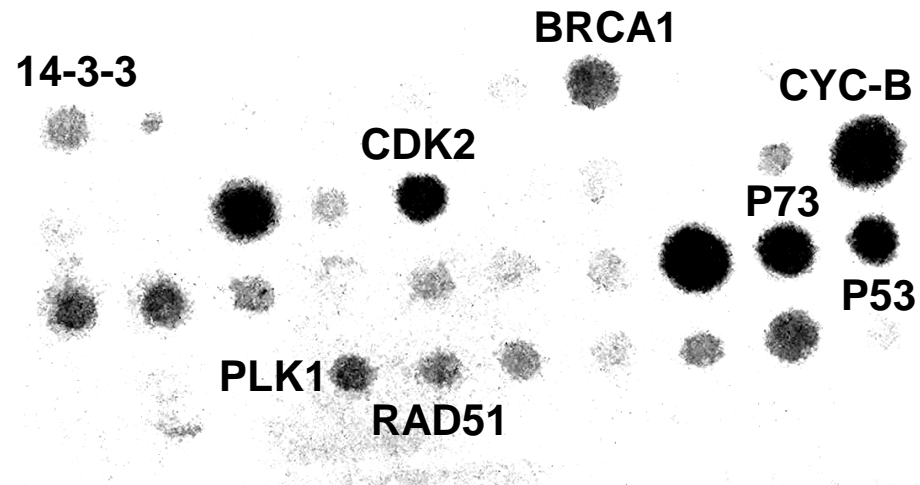

## B APEX2-interacting partners

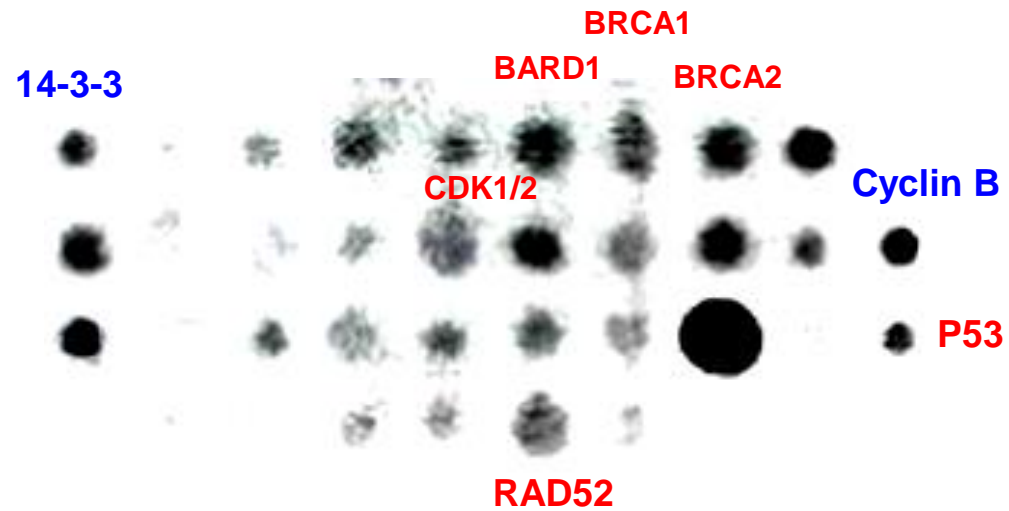

Supplement: Supplementary file 5 — Supplementary Figure 4 [file 41408_2018_129_MOESM5_ESM.pdf]

**DMSO**

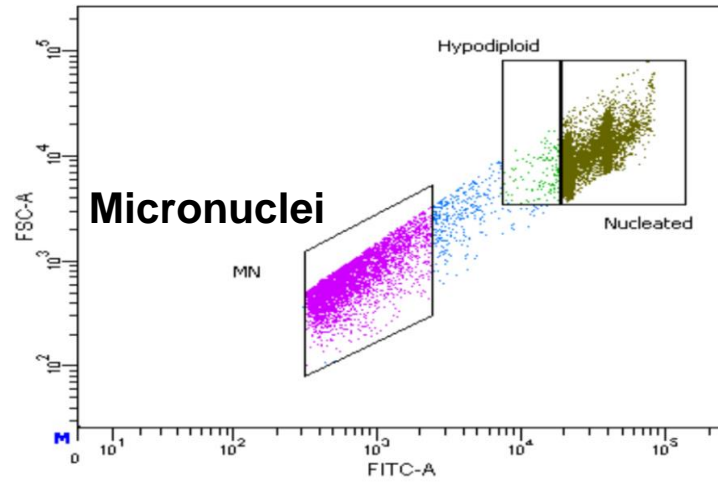

**API-III**

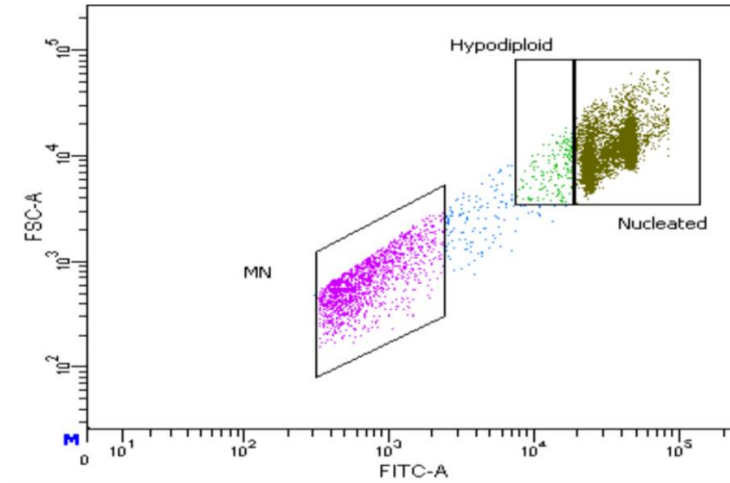

**Melphalan**

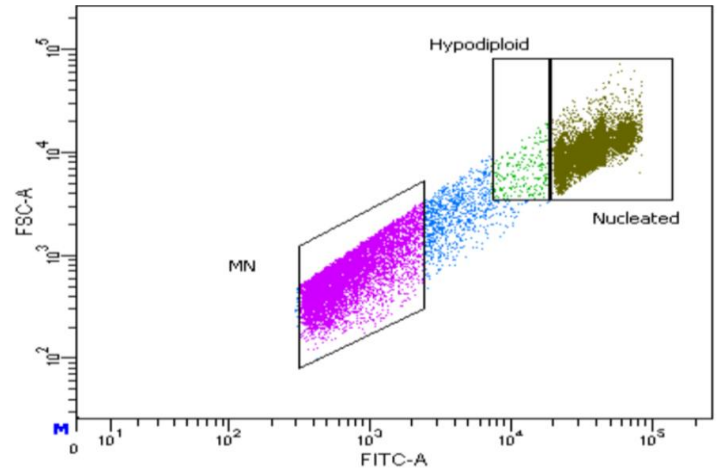

**API-III+Melphalan**

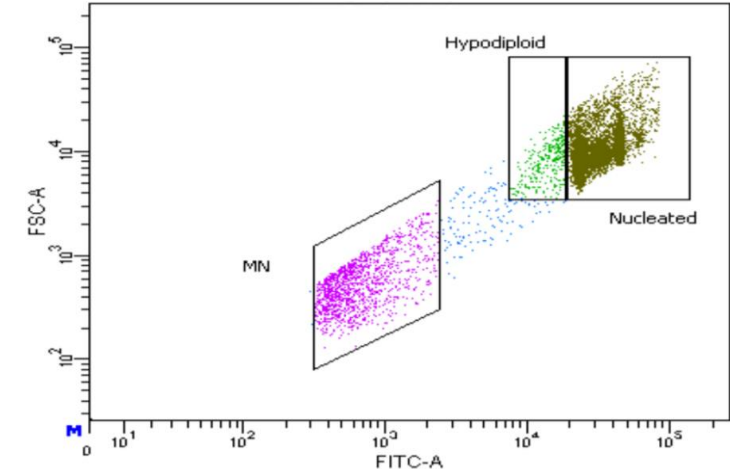

Supplement: Supplementary file 6 — Supplementary Figure 5 [file 41408_2018_129_MOESM6_ESM.pdf]
